# Profiling migraine patients according to clinical and psychophysical characteristics: clinical validity of distinct migraine clusters

**DOI:** 10.1007/s10072-023-07118-8

**Published:** 2023-10-13

**Authors:** Stefano Di Antonio, Lars Arendt-Nielsen, Marta Ponzano, Francesca Bovis, Paola Torelli, Pelosin Elisa, Cinzia Finocchi, Matteo Castaldo

**Affiliations:** 1 Department of Health Science and Technology, Center for Pain and Neuroplasticity (CNAP), School of Medicine, SMI, Aalborg University, Aalborg, Denmark; 2 Department of Neuroscience, Rehabilitation, Ophthalmology, Genetics and Maternal Child Health, Genoa, Italy; 3 Department of Gastroenterology & Hepatology, Mech-Sense, Clinical Institute, Aalborg University Hospital, 9000 Aalborg, Denmark; 4 Clinical Institute, Steno Diabetes Center North Denmark, Aalborg University Hospital, 9000 Aalborg, Denmark; 5 Department of Health Sciences (DISSAL), Section of Biostatistics, University of Genoa, Genoa, Italy; 6 Headache Centre, Department of Medicine and Surgery, University of Parma, Parma, Italy; 7 IRCCS, Ospedale Policlinico San Martino, Genoa, Italy; 8 Ospedale San Paolo, ASL 2 Savonese, Savona, Italy

**Keywords:** Migraine, Precision medicine, Phenotypes, Quantitative sensory testing, Cervical musculoskeletal impairments

## Abstract

**Aims:**

Investigate if different clinical and psychophysical bedside tools can differentiate between district migraine phenotypes in ictal/perictal (cohort 1) and interictal (cohort 2) phases.

**Method:**

This observational study included two independent samples in which patients were subgrouped into distinct clusters using standardized bedside assessment tools (headache frequency, disability, cervical active range of motion, pressure pain threshold in different areas): (A) cohort 1—ictal/perictal migraine patients were subgrouped, based on previous studies, into two clusters, i.e., Cluster-1.1 No Psychophysical Impairments (NPI) and Cluster-1.2 Increased Pain Sensitivity and Cervical Musculoskeletal Dysfunction (IPS-CMD); (B) cohort 2—interictal migraine patients were subgrouped into three clusters, i.e., Cluster-2.1 NPI, Cluster-2.2 IPS, and Cluster-2.3 IPS-CMD. Clinical characteristics (multiple questionnaires), somatosensory function (comprehensive quantitative sensory testing (QST)), and cervical musculoskeletal impairments (cervical musculoskeletal assessment) were assessed and compared across headache clusters and a group of 56 healthy controls matched for sex and age.

**Results:**

Cohort 1: A total of 156 subjects were included. Cluster-1.2 (IPS-CMD) had higher headache intensity (*p* = 0.048), worse headache-related (*p* = 0.003) and neck-related disability (*p* = 0.005), worse quality of life (*p* = 0.003), and higher symptoms related to sensitization (*p* = 0.001) and psychological burden (*p* = 0.005) vs. Cluster-1.1(NPI). Furthermore, Cluster-1.2 (IPS-CMD) had (1) reduced cervical active and passive range of motion (*p* < 0.023), reduced functionality of deep cervical flexors (*p* < 0.001), and reduced values in all QST(*p* < 0.001) vs. controls, and (2) reduced active mobility in flexion, left/right lateral flexion (*p* < 0.045), and reduced values in QST (*p* < 0.001) vs. Cluster-1.1 (NPI). Cohort 2: A total of 154 subjects were included. Cluster-2.3 (IPS-CMD) had (1) longer disease duration (*p* = 0.006), higher headache frequency (*p* = 0.006), disability (*p* < 0.001), and psychological burden (*p* = 0.027) vs. Cluster-2.2 (IPS) and (2) higher headache-related disability (*p* = 0.010), neck-related disability (*p* = 0.009), and higher symptoms of sensitization (*p* = 0.018) vs. Cluster-2.1 (NPI). Cluster-2.3(IPS-CMD) had reduced cervical active and passive range of motion (*p* < 0.034), and reduced functionality of deep cervical flexors (*p* < 0.001), vs. controls, Custer-2.1 (NPI), and Cluster-2.2 (IPS). Cluster-2.2 (IPS) and 2.3 (IPS-CMD) had reduced QST values vs. controls (*p* < 0.001) and Cluster-2.1 (*p* < 0.039).

**Conclusion:**

A battery of patient-related outcome measures (PROMs) and quantitative bedside tools can separate migraine clusters with different clinical characteristics, somatosensory functions, and cervical musculoskeletal impairments. This confirms the existence of distinct migraine phenotypes and emphasizes the importance of migraine phases of which the characteristics are assessed. This may have implications for responders and non-responders to anti-migraine medications.

## Introduction

Migraine is a common neurovascular brain disorder considered among the primary causes of disability worldwide [1], and no significant improvements were made in its burden between 1990 and 2016 [1, 2]. Thus, in recent years, researchers put great effort into developing a new class of drugs specifically targeting the CGRP peptide or receptors that could reduce the migraine burden [3]. However, this new therapy is known not to be efficient in all migraine patients [4]. Moreover, due to its high cost [5], this new class of drugs are not considered the first treatment approach in migraine management [6]. To enhance the cost-effectiveness of migraine treatment and reduce the burden of this disease, precision medicine principles should be applied clinically in migraine patients [7]. Precision medicine requires the use of various biomarkers to identify distinct phenotypes within the same medical condition with the aim to identify responders and non-responders to different treatment approaches [8]. In migraine patients, psychophysical characteristics is one parameter to be used to profile patients in different subgroups and predict treatment response [9–12]. The biomarkers used to profile migraine patients often involve time-consuming assessment that requires expensive tools [9–12], not always available or applicable in a clinical setting. We have recently applied fourteen clinical patient–related outcome measures (PROMs) and psychophysical bedside tools used in a clinical setting to profile and identify subgroups of migraine patients [13]. Episodic and chronic migraine patients were assessed in the ictal/perictal phase and interictal phase. In the ictal/perictal phase, two distinct clusters were identified, with one group showing no psychophysical impairment, and one showing increased pain sensitivity and cervical musculoskeletal dysfunctions, as well as higher disability. In the interictal phase, three distinct clusters could be identified, with one group showing no psychophysical impairment, one increased pain sensitivity, and one increased pain sensitivity and cervical musculoskeletal dysfunctions, as well as higher headache frequency, and disability [13].

However, as a limited number of clinical and psychophysical bedside tools were used to identify these distinct subgroups, the clinical validity should be further investigated before assuming these subgroups could be considered clinically different migraine phenotypes. The aim of this paper was to investigate the clinical importance of the distinct migraine subgroups by (1) assessing differences in clinical characteristics, including psychological burden, using a core set of PROMs and (2) assessing quantitative differences in somatosensory function and cervical musculoskeletal impairments using comprehensive quantitative sensory testing and cervical musculoskeletal assessment. These paper results will add important information to our previous finding [13] as the distinct migraine subgroups will be also compared to a control group of healthy subjects, and multiple clinical, psychological, and psychophysical characteristics will be assessed. The assessment of migraineur illness perception and psychological aspects using variously subjective measures (PROMs) together with objective measures of migraine psychophysical manifestation (quantitative sensory testing and cervical musculoskeletal assessment) will give important insights regarding the interaction between objective signs and subjective experience of the disease [14]. These results will help clinicians to tailor a more personalized treatment approach following the biopsychosocial model [14].

## Method

### Design

This multicenter, cross-sectional, observational study was conducted at the Parma and Genova Headache Center and approved by the Ligurian (244/2018) and “Area Vasta Emilia-Nord” (18,305/2019) regional ethic committee. All subjects signed an informed consent form and were assessed between April 2019 and February 2022. This study was based on two cohorts of migraine patients that have previously been the basis for a recent paper [13].

### Population

Patients on waiting lists to receive their first visit to the Headache Center were invited to participate in this study. Men and women aged between 18 and 65 with episodic (EM) or chronic (CM) migraine with or without aura for at least 3 months were included. As psychophysical characteristics vary according to the migraine phase [15, 16], patients were divided into two distinct cohorts according to the migraine phase in which the psychophysical examination was performed.

In cohort 1, ictal/perictal EM and CM were included. EM patients were considered in the ictal phase if they had a headache during the visit and in the perictal phase if they have a headache within the 24 h before or after the visit [17, 18]. CM patients were considered in the ictal phase if they had any type of headache (with tension-type or migraine characteristics) during the visit. Patients were excluded if they had any other primary/secondary headache; less than 1 headache attack in 4 weeks; changes of headache characteristics, or onset of a “new” headache after COVID-19 infection/vaccination; any other neurologic, psychiatric, rheumatologic, or systemic pathology with medical diagnosis; history of head/neck trauma in the previous year; received cervical/head surgery; received manual therapy in the cervical spine, or cervical anesthetic block, or botulin injection in the last 6 months; changed the prophylactic treatment in the last 3 months; or were unable to speak and understand Italian.

In cohort 2, interictal EM and CM were included. EM patients were considered in the interictal phase if they were headache-free during the visit and did not have a headache within the 24 h before or after the visit [17, 18]. CM patients were considered in the interictal phase if they were headache-free during the visit. The exclusion criteria were the same used for ictal/perictal patients (cohort 1) with the exception that patients who used acute pharmacologic treatment in the 24 h before the assessment were excluded.

Control participants were recruited specifically for this study. They were defined as healthy subjects with a maximum of two headache episodes per year that did not fulfill the criteria for migraine or any other primary headache type with no family history of migraine or other primary headaches. The exclusion criteria for the control subjects were the same as the criteria used for migraine patients in cohort 1.

### Procedure

The first screening was made by telephone interview in which patients were excluded if they presented any signs of red flags [19], or any exclusion criteria. Healthy controls were recruited from university students, hospital staff and university staff, and the general population through print and social media advertising. Then, a physical examination was performed in which one physiotherapist for each recruitment center (S.D., M.C.), blinded to the presence of headache, performed the assessment (psychophysical examination, questionnaire, and explanation of how to fulfill a diary for the following 4 weeks) and recorded the interval between the assessment and the last headache attack. Four weeks following the first evaluation, patients were visited by a neurologist who performed a diagnosis of headache according to the ICHD-3 [20]‚ and CM and EM patients with or without aura were included and divided into two cohorts that had undergone two separate analyses according to our previous suggestion [13].

Cohort 1: EM and CM in the ictal/perictal phase; cohort 2: EM and CM in the interictal phase. In each cohort, migraine patients were profiled into different clusters according to clinical and psychophysical characteristics [13]:

Cohort 1:

- Cluster-1.1: Migraine with no psychophysical impairments (NPI)
- Cluster-1.2: Migraine with increased pain sensitivity and cervical musculoskeletal dysfunctions (IPS-CMD)

Cohort 2:

- Cluster-2.1: Migraine with no psychophysical impairments (NPI)
- Cluster-2.2: Migraine with increase pain sensitivity (IPS)
- Cluster-2.3: Migraine with increased pain sensitivity and cervical musculoskeletal dysfunctions (IPS-CMD)

### Assessments

For each subject, general characteristics were assessed (Table 1). Migraine patients used a daily updated diary recording the total use of drugs and the frequency, intensity, and duration of headache attacks. Moreover, the headache side and total years lived with the headache were recorded (Table 2).

**Table 1:** General characteristics

	Controls	Ictal/perictal M		Interictal M		
	Controls (56)	Cluster-1.1 NPI (19)	Cluster-1.2 IPS-CMD (81)	Cluster-2.1 NPI (18)	Cluster-2.2 IPS (44)	Cluster-2.3 IPS-CMD (36)
General characteristics						
Age, mean years (SD)	37.2 (14.3)	40.9 (12.9)	39.0 (12.0)	36.9 (13.2)	31.2 (10.0)	43.4 (9.2)
BMI, mean (SD)	22.1 (2.7)	23.9 (4.1)	23.5 (4.0)	22.3 (3.3)	22.1 (3.7)	23.6 (3.6)
Gender, *N* (%)						
Female	40 (71%)	13 (68%)	72 (89%)	8 (44%)	36 (82%)	30 (83%)
Male	16 (29%)	6 (32%)	9 (11%)	10 (56%)	8 (18%)	6 (17%)
Acute treatment 24 h before the evaluation, *N* (%)						
Yes	1 (2%)	6 (32%)	16 (20%)	0 (0%)	0 (0%)	0 (0%)
No	55 (98%)	13 (68%)	65 (80%)	18 (100%)	44 (100%)	36 (100%)

*BMI* body mass index, *IPS-CMD* increased pain sensitivity and cervical musculoskeletal dysfunctions, *IPS* increased pain sensitivity, *NPI* no psychophysical impairments, *N* number, *SD* standard deviation

**Table 2:** Difference in general, clinical, and psychophysical characteristics across M patients

a Ictal/perictal M (cohort 1)								
		Cluster-1.1 NPI		Cluster-1.2 IPS-CMD		Between group difference		
Years lived with headache, median (25th–75th)^‡^		16 (11–34)		14 (3–28)		*U* = 607.9, *p* = 155		
Frequency, median day/4 weeks (25th–75th)^‡^		6 (5–10)		9 (5.5–12.5)		*U* = 613.0, *p* = 0.170		
Intensity, median NPRS 0–10 (25th–75th)^‡^		4.3 (2.8–6)		5.5 (4.1–7.1)		*U* = 544.0, *p* = 0.048*		
Duration, median hours/day (25th–75th)^‡^		6.1 (3.3–9.8)		7.3 (4.6–10)		*U* = 688.0, *p* = 0.478		
Use of drugs, median (25th–75th)^‡^		3 (2–8)		5 (3–8.5)		*U* = 650.5, *p* = 0.297		
Headache side, *N* (%)^#^						*χ*^2^ < 0.1, *p* = 0.978		
Bilateral/side shift		16 (84%)		68 (84%)				
Unilateral (left or right)		3 (16%)		13 (16%)				
Presence of neck pain, *N* (%)^#1^						*χ*^2^ = 6.4, *p* = 0.012*		
Yes		8 (42%)		58 (73%)				
No		11 (58%)		22 (27%)				
HDI-P, mean (SD)^§^		21.8 (10.4)		25.8 (10.5)		*t* = − 1.4, *p* = 0.138		
HDI-E, median (25th–75th)^‡^		12 (8–18)		24 (12–32)		*U* = 433.0, *p* = 0.003*		
NDI, median (25th–75th)^‡1^		18 (6–22)		24 (16–36)		*U* = 451.0, *p* = 0.005*		
NDI-physical, median (25th–75th)^‡1^		6.7 (0–16.7)		8.5 (16.7–30)		*U* = 448.5, *p* = 0.005*		
NDI-mental, median (25th–75th)^‡1^		25 (15–40)		35 (25–50)		*U* = 507.0, *p* = 0.023*		
SF-36, physical, median (25th–75th)^‡2^		71.9 (55–78.1)		54.4 (41.3–70.6)		*U* = 406.5, *p* = 0.003*		
SF-36 mental, median (25th–75th)^‡2^		69.4 (53.7–78.3)		58.3 (40.1–74.4)		*U* = 525.5, *p* = 0.068		
CSI, mean (SD)^§^		28.7 (14.2)		39.7 (11.9)		*t* = − 3.5, *p* = 0.001*		
HADS-A, mean (SD)^§3^		5.4 (4.6)		8.3 (3.8)		*t* = − 2.9, *p* = 0.005*		
HADS-D, median (25th–75th)^‡3^		2 (1–5)		5 (3–8)		*U* = 446.5, *p* = 0.005*		
b Interictal M (cohort 2)								
	Cluster-2.1 NPI	Cluster-2.2 IPS	Cluster-2.3 IPS-CMD		Between group difference	NPI vs IPS	NPI vs IPS-CMD	IPS vs IPS-CMD
Years lived with headache, median (25th–75th)^‡‡^	14 (7.8–29.5)	8 (4.3–15.8)	19.5 (7–30.5)		*χ*^2^ = 10.1 *p* = 0.006*	*p* = 0.216	*p* = 1.000	*p* = 0.006*
Frequency, median day/4 weeks (25th–75th)^‡^^‡^	5.5 (4–10.5)	5 (3.3–7)	8.5 (4–11)		*χ*^2^ = 8.8 *p* = 0.012*	*p* = 1.000	*p* = 1.000	*p* = 0.006*
Intensity, mean NPRS 0–10^§§^	6.1(1.5)	5.4(1.7)	5.9(1.7)		*F* = 1.6, *p* = 0.201	*p* = 0.314	*p* = 1.000	*p* = 0.584
Duration, median hours/day (25th–75th)^‡^^‡^	5.5 (3.7–10)	6.2 (3.4–10.9)	6.9 (3.2–10)		*χ*^2^ = 3.4 *p* = 0.842	*p* = 1.000	*p* = 1.000	*p* = 1.000
Use of drugs, median (25th–75th)^‡‡^	4 (1.5–7.5)	3 (2–5)	5.5 (3–9.8)		*χ*^2^ = 8.7, *p* = 0.013*	*p* = 1.000	*p* = 0.519	*p* = 0.009*
Headache side, N (%)^#^								
Bilateral/side shift	14 (78%)	33 (75%)	22 (61%)		*χ*^2^ = 2.4, *p* = 0.300	*p* = 1.000	*p* = 0.663	*p* = 0.546
Unilateral (left or right)	4 (22%)	11 (25%)	14 (40%)					
Presence of neck pain, N (%)^#4^					*χ*^2^ = 4.9, *p* = 0.085			
Yes	7 (39%)	22 (50%)	24 (69%)					
No	11(61%)	22(50%)	11(31%)					
HDI-P, mean (SD)^§§^	21.8 (8.6)	20.3 (8.8)	27.4 (10.3)		*F* = 6.1, *p* = 0.003*	*p* = 1.000	*p* = 0.855	*p* = 0.003*
HDI-E, mean (SD)^§§^	15.6 (8.0)	15.9 (9.6)	23.6 (9.2)		*F* = 8.2, *p* = 0.001*	*p* = 1.000	*p* = 0.010*	*p* < 0.001*
NDI, median (25th–75th)^‡‡4^	11 (8–18.5)	14 (8.5–22)	22 (12–30)		*χ*^2^ = 7.0 *p* = 0.030*	*p* = 1.000	*p* = 0.093	*p* = 0.066
NDI-physical, median (25th–75th)^‡‡4^	3.3 (0–10)	6.7 (3.3–13.3)	13.3 (6.7–23.3)		*χ*^2^ = 10.0 *p* = 0.007*	*p* = 0.702	*p* = 0.009*	*p* = 0.057
NDI-mental, mean (SD)^§§4^	26.9 (13.4)	25.5 (15.0)	31.3 (14.7)		*F* = 1.6, *p* = 0.211	*p* = 1.000	*p* = 0.933	*p* = 0.247
SF-36, physical median (25th–75th)^‡‡5^	76.9 (53.6–88)	71.9 (55.3–83.1)	66.3 (40–79.7)		*χ*^2^ = 4.4, *p* = 0.112	*p* = 1.000	*p* = 0.201	*p* = 0.261
SF-36 mental median (25th–75th)^‡‡5^	70.7 (49.2–85.8)	69.6 (53.5–81.2)	58.7 (48.3–78.4)		*χ*^2^ = 3.0 *p* = 0.225	*p* = 1.000	*p* = 0.516	*p* = 0.393
CSI, mean (SD)^§§^	28.2 (13.3)	32.5 (12.7)	38.4 (12.1)		*F* = 4.5, *p* = 0.014*	*p* = 0.674	*p* = 0.018*	*p* = 0.116
HADS-A, median (25th–75th)^‡‡4^	5.5 (2–9.5)	6 (3–9)	7 (5–10)		*χ*^2^ = 2.8, *p* = 0.245	*p* = 1.000	*p* = 0.630	*p* = 0.387
HADS-D, median (25th–75th)^‡‡4^	2 (0.8–5.3)	2.5 (1–5)	5 (2–7)		*χ*^2^ = 7.8, *p* = 0.020*	*p* = 1.000	*p* = 0.126	*p* = 0.027*

*CSI* Central Sensitization Inventory, *HADS-A* Hospital Anxiety and Depression Scale Anxiety, *HADS-D* Hospital Anxiety and Depression Scale Depression, *HDI-E* Headache Disability Index Emotional, *HDI-P* Headache Disability Index Physical, *IPS-CMD* increased pain sensitivity and cervical musculoskeletal impairment, *IPS* increased pain sensitivity, *NDI* Neck Disability Index, *NPI* no psychophysical impairments, *NPRS* Numeric Pain Rating Scale, *N* number, *SD* standard deviation, *SF-36* Medical Outcomes Study Short Form 36

^§^*T*-test

^‡^Mann–Whitney test

^#^Chi-square test

^§§^ANOVA with Bonferroni post hoc analyses

^‡‡^Kruskal–Wallis with the Mann–Whitney test post hoc (Bonferroni corrected *p*-value)

^*^Significant at *p* < 0.05; normality was assessed with Shapiro–Wilk test

^1^Due to missing data 80 patients were included in Cluster-1.2

^2^Due to missing data 76 patients were included in Cluster-1.2

^3^Due to missing data 79 patients were included in Cluster-1.2

^4^Due to missing data 35 patients were included in Cluster-2.3

^5^Due to missing data 33 patients were included in Cluster-2.3

#### Questionnaires

- *Headache disability index* (*HDI*): The HDI questionnaire was used to assess the emotional (HDI-E 0–52) and the physical (HDI-P 0–48) headache-related disability. The higher the score, the higher the headache-related disability [21].
- *Neck disability index* (*NDI*): Neck-related disability was assessed using the NDI (0–100%), and the total score was calculated [22]. Then the subscale regarding physical function (personal care, lifting, work, driving, sleeping, and recreation; NDI-physical 0–100%) and mental function (neck pain, reading, headaches, and concentration; NDI-mental 0–100%) were calculated [23]. The higher the score, the higher the neck-related disability. NDI questionnaires were also used to identify patients with or without the presence of neck pain [16, 24].
- *Medical Outcomes Study Short Form 36* (*SF-36*): The SF-36 questionnaire was used to assess two components of quality of life: the physical health dimension (SF-36 physical, total score 0–100) and the mental health dimension (SF-36 emotional, total score 0–100). The lower the score, the lower the quality of life [25].
- *Central sensitization inventory* (*CSI*): The CSI questionnaire was used to assess symptoms related to sensitization. The higher the score, the more symptoms related to central sensitization are present (0–100). A cutoff value of 40 is normally used in the literature [26].
- *Hospital Anxiety and Depression Scale* (*HADS*): The HADS was used to assess the impact of anxiety (HADS-A) and depressive (HADS-D) symptoms. A higher score indicates a higher level of anxiety and/or depression (HADS: 0–21; HADS-D: 0–21) [27].

#### Psychophysical characteristics: cervical musculoskeletal impairments

Physical examination tests were used to assess the presence of cervical musculoskeletal impairments and were differentiated into tests aimed to evaluate cervical musculoskeletal functionality (cervical musculoskeletal dysfunction) and test aimed to identify the presence of referred pain [16].

#### Cervical musculoskeletal dysfunctions

- *Active range of motion* (*AROM*): Cervical AROM (extension, flexion, left/right lateral flexion, left/right rotation) was recorded in degrees of movement with the cervical range of motion (CROM) device [28].
- *Flexion rotation test* (*FRT*): Passive mobility of the upper cervical spine was assessed in degree with FRT. The subject was supine on the couch, and the therapist passively moved his neck to end-range cervical flexion. In this flexed position, the head and neck were passively rotated as far as possible within comfortable limits to the left and then to the right. The total range of motion during the FRT (sum left and right, °) was calculated in degrees using the CROM device [29].
- *Craniocervical flexion test* (*CCFT*): The craniocervical flexion test assessed the function of deep cervical flexors muscles. A pressure biofeedback unit 20–30 mmHg was used. Subjects performed craniocervical flexion in five incremental stages (one stage every 2 mmHg), and the mmHg value that was held for 10 s without compensation was recorded as the activation pressure score (APS) [30].

#### Referred pain

- *Passive accessory intervertebral movements* (*PAIVMs*): The number of positive vertebral segments (pain referred to the head region in control, typical migraine pain reproduction in patients) was assessed with PAIVMs over to C-0/C-1 and C-2/C-3 vertebral segments bilaterally [31].
- *Myofascial trigger points* (*MTrPs*): MTrPs were defined as a hypersensitive spot in a taut band in the muscle belly that resulted in a referred pain, either recognized by the patient as the usual headache (active MTrPs) or referred into a non-headache region (latent MTrPs). The presence of MTrPs was assessed bilaterally in the temporal muscles, masseter muscle, sternocleidomastoid muscle, suboccipital muscles, splenius muscles, and trapezius muscle. The total number of active and latent trigger points was recorded [32].

### Psychophysical characteristics: somatosensory function

Quantitative sensory testing (QST) was performed from distal pain-free areas first, then the cervical area, and finally the trigeminal area (symptomatic side in patients with unilateral migraine; dominant side in patients with side/shift or bilateral migraine and in controls) to assess signs related to sensitization [15]:

- *Static pressure pain threshold* (*sPPT*): sPPT to hand-held electronic algometry (Somedic-AB, Sweden), probe area 1cm^2^, 30 kPa/s force increase)) [15] was assessed over the trigeminal area (temporalis muscle), upper cervical spine (left and right articular pillars of C1 and C2 vertebral segments), lower cervical spine (left and right articular pillars of C4 and C6 vertebral segments); distal pain-free areas (second metacarpophalangeal joint of the dominant hand; tibialis anterior muscle of the dominant leg).
- *Dynamic pressure pain threshold* (*dPPT*): dPPT to a dynamic algometry (constant force spring controlled from 550 to 5300 g) was assessed over the posterior aspect of the neck (left and right sides) [15].
- *Mechanical pain threshold* (*MPT*): MPT to pinprick stimulation (from 0.80 to 50.1 g metal probes) was assessed over the trigeminal area (temporalis muscle) and distal pain-free areas (thenar eminence of the dominant hand) [15].

For sPPT, dPPT, and MPT, the lower the threshold, the more sensitization:

• *Wind-up ratio* (*WUR*): The WUR was calculated to assess the temporal summation of mechanical pain. The patient was given a pain rating (11-point numeric rating scale) for the first and last stimulus of 10 stimuli given with pinprick over temporalis (50.1 g). The difference between the pain rating of the last of a ten stimuli series and the first stimulus was calculated (WUR) [15]. A positive WUR was a sign of increased temporal summation of pain, and the higher the WUR, the more the sensitization.

Details of the assessment were previously presented [15, 16, 33].

### Statistical analysis

The sample size was calculated using G*Power 3.1. To achieve a moderate/large effect size (*f*: 0.35) with a power of 85% and an alpha level of 0.05 with a general linear model using 4 covariates and 3 groups (cohort 1), 131 subjects were required. In cohort 2, where 4 covariates and 4 groups were used, 139 subjects were required [34].

Differences across migraine groups in clinical characters were investigated with the *t-*test, Mann–Whitney test, or the chi-square test according to variable type and distribution when two migraine clusters were present. When more than two clusters were present, differences in general characters and frequency were investigated with the ANOVA, Kruskal–Wallis test, or chi-square test, according to variable type and distribution. The Bonferroni, the Mann–Whitney test, and the chi-square test were used to run post hoc analyses respectively for ANOVA, the Kruskal–Wallis test, and the chi-square test using a Bonferroni corrected *p*-value.

Differences in psychophysical characteristics across migraine groups and controls were investigated by transforming non-normal distributed variables to fulfill the normality assumption (the normality of the data was assessed with the Shapiro–Wilk test). The same sample of healthy controls was used as comparison both in cohort 1 and in cohort 2 analysis. To avoid that differences in general characteristics could bias the results, a general linear model was performed, including age, gender, body mass index, and use of acute pharmacological treatment in the 24 h before the evaluation as covariates in the models. A Bonferroni-adjusted post hoc analysis was performed to make single-group comparisons when more than two clusters were compared. Subjects with any missing data were excluded from the analysis in which data were not available. The threshold accepted for the statistical significance of the results was *p* < 0.05, and tests of statistical significance were two-tailed. Statistical analyses were performed using the SPSS software (version 24).

## Results

**Fig. 1:**
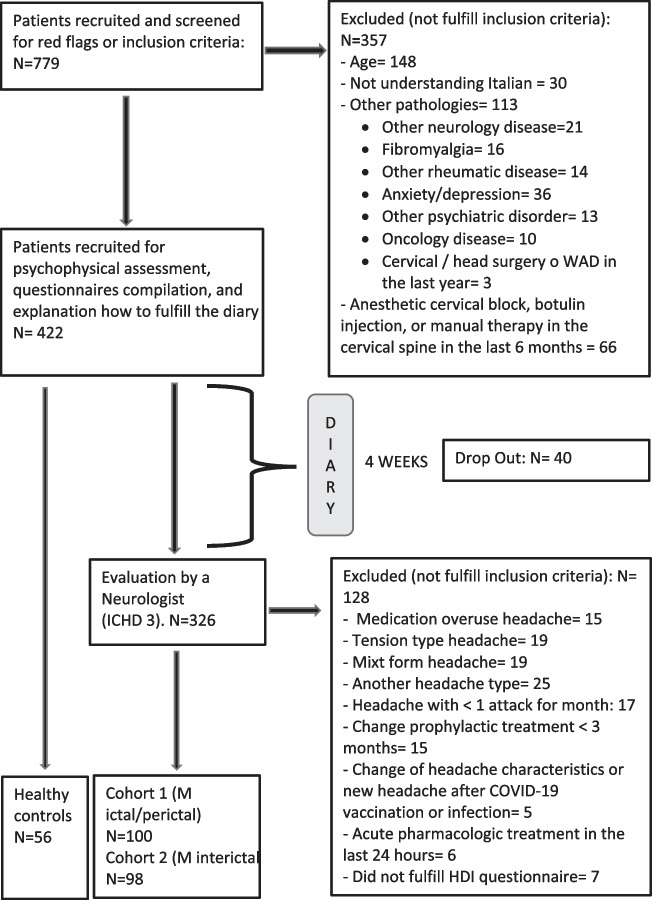
Flow chart. HDI, headache disability index; ICHD, international classification headache disorders; M, migraine; N, number

A total of 779 subjects were initially screened. A total of 56 healthy subjects, 100 migraine patients assessed in the ictal/perictal phase (cohort 1), and 98 migraine patients assessed in the interictal phase (cohort 2) were included (Fig. 1). The general characteristics of the population included are reported in Table 1.

### Cohort 1: ictal/perictal migraine patients

#### Clinical characteristics

Compared to Cluster-1.1 (NPI), Cluster-1.2 (IPS-CMD) had higher headache intensity (*p* = 0.048), worse HDI-E (*p* = 0.003), NDI (*p* = 0.005), NDI-physical (*p* = 0.005), NDI-mental (*p* = 0.023), SF-36 physical (*p* = 0.003), CSI (*p* = 0.001), HADS-A (*p* = 0.005), HADS-D (*p* = 0.005), and higher percentage of patients with neck pain (*p* = 0.012). No other differences were observed (Table 2a).

#### Psychophysical characteristics

Cluster-1.2 (IPS-CMD) had the following:

- Compared to controls: reduced AROM in flexion, extension, right and left lateral flexion, right and left rotation, FRT, APS, and higher number of MTrPs and PAIVMs (all, *p* < 0.023). Higher trigeminal WUR (*p* = 0.019), lower trigeminal MPT (5.7(7.3) g vs 22.1(18.5) g) and sPPT, sPPT over upper and lower cervical spine, cervical dPPT, hand sPPT and MPT, and leg sPPT (all, *p* < 0.001).
- Compared to Cluster-1.1 (NPI): reduced AROM in flexion, right and left lateral flexion, and higher number of MTrPs and PAIVMs (all, *p* = 0.045). Lower trigeminal, upper and lower cervical sPPT, cervical dPPT, hand and leg sPPTs (all, *p* < 0.001).

Cluster-1.1 (NPI) had compared to controls the following:

• Reduced APS and higher number of MTrPs and PAIVMs (all, *p* < 0.001). Higher trigeminal WUR (*p* = 0.028) (higher sensitization) and higher sPPT over temporalis, upper cervical spine, and leg (lower sensitization) (all, *p* < 0.049).

No other differences were observed (Table 3a).

**Table 3:** A general linear models (GLM) including age, gender, body mass index, and use of acute pharmacological treatment in the 24 h before the evaluation as covariates in the model was performed to assess differences across groups. Bonferroni-adjusted post hoc analyses was performed to make single groups comparisons

a Ictal/perictal M (cohort 1)													
	Controls (56)		Cluster-1.1 NPI (19)	Cluster-1.2 IPS-CMD (81)		Between group difference		Control vs NPI		Control vs IPS-CMD		NPI vs IPS-CMD	
Cervical musculoskeletal impairments													
AROM flexion, mean ° (SD)^‡^	62.9 (9.7)		61.3 (9.0)	51.1 (12.1)		*F* = 18.5, *p* < 0.001*		*p* = 1.000		*p* < 0.001*		*p* < 0.001*	
AROM extension, mean ° (SD)	72.9 (15.5)		68.6 (17.9)	61.1 (16.2)		*F* = 9.4, *p* < 0.001*		*p* = 1.000		*p* < 0.001*		*p* = 0.054	
AROM right lateral flexion, mean ° (SD)^‡^	40.0 (9.5)		44.1 (14.5)	33.9 (9.7)		*F* = 11.1, *p* < 0.001*		*p* = 0.294		*p* = 0.004*		*p* < 0.001*	
AROM left lateral flexion, mean ° (SD)	46.1 (9.5)		46.2 (13.6)	40.4 (11.6)		*F* = 5.3, *p* = 0.006*		*p* = 1.000		*p* = 0.023*		*p* = 0.045*	
AROM right rotation, mean ° (SD) ^‡^	68.6 (8.6)		63.5 (14.6)	61.8 (12.1)		*F* = 6.0, *p* = 0.008*		*p* = 0.925		*p* = 0.006*		*p* = 0.709	
AROM left rotation, mean ° (SD) ^‡^	69.6 (8.5)		66.0 (14.2)	62.4 (11.9)		*F* = 5.6, *p* = 0.005*		*p* = 1.000		*p* = 0.005*		*p* = 0.253	
FRT, total, mean° (SD)^‡^	102.2 (11.2)		90.2 (16.2)	82.9 (19.5)		*F* = 18.4, *p* < 0.001*		*p* = 0.180		*p* < 0.001*		*p* = 0.064	
APS, mean mmHg (SD) ^§^	27.7 (2.8)		22.3 (2.5)	22.9 (3.0)		*χ*^2^ = 95.2, *p* < 0.001*		*p* < 0.001*		*p* < 0.001*		*p* = 1.000	
Total MTrPs, mean number (SD) ^‡^	3.9 (3.6)		6.7 (4.1)	9.1 (3.2)		*F* = 44.2, *p* < 0.001*		*p* = 0.001*		*p* < 0.001*		*p* = 0.024*	
Total PAIVMs, mean number (SD) ^§^	0.9 (1.4)		2.1 (1.6)	3.3 (1.3)		*χ*^2^ = 95.4, *p* < 0.001*		*p* < 0.001*		*p* < 0.001*		*p* = 0.015*	
Quantitative sensory testing													
WUR temporalis, mean (SD)^†^	1.4 (2.1)		2.7 (2.8)	2.7 (2.3)		*F* = 5.1, *p* = 0.007*		*p* = 0.028*		*p* = 0.019*		*p* = 1.000	
MPT temporalis, mean g (SD)^†^	22.1 (18.5)		15.9 (19.5)	5.7 (7.3)		*F* = 20.8, *p* < 0.001*		*p* = 0.086		*p* < 0.001*		*p* = 0.073	
sPPT temporalis, mean kPa (SD)^†^	248.3 (98.1)		318.1 (88.5)	144.3 (50.5)		*F* = 50.4, *p* < 0.001*		*p* = 0.040*		*p* < 0.001*		*p* < 0.001*	
sPPT UCS total, mean kPa (SD)^†^	511.0 (232.3)		667.8 (197.4)	311.5 (108.6)		*F* = 41.3, *p* < 0.001*		*p* = 0.049*		*p* < 0.001*		*p* < 0.001*	
sPPT LCS total, mean kPa (SD)^†^	605.2 (263.5)		793.9 (313.4)	349.1 (128.1)		*F* = 42.5, *p* < 0.001*		*p* = 0.102		*p* < 0.001*		*p* < 0.001*	
dPPT total mean g (SD)^†^	7766.1 (2779.6)		6692.1 (3037.4)	3706.2 (2574.7)		*F* = 41.4, *p* < 0.001*		*p* = 0.265		*p* < 0.001*		*p* < 0.001*	
sPPT second MCP, mean kPa (SD)^†^	328.2 (135.9)		410.1 (132.6)	242.2 (86.8)		*F* = 18.7, *p* < 0.001*		*p* = 0.130		*p* < 0.001*		*p* < 0.001*	
MPT thenar eminence, mean g (SD)^†^	32.3 (16.1)		27.1 (17.3)	22.4 (16.1)		*F* = 6.5, *p* = 0.001*		*p* = 0.530		*p* < 0.001*		*p* = 0.747	
sPPT tibialis muscle, mean kPa (SD)^†^	438.3 (211.4)		587.6 (206.0)	300.4 (126.0)		*F* = 22.4, *p* < 0.001*		*p* = 0.007*		*p* < 0.001*		*p* < 0.001*	
b Interictal M (cohort 2)													
	Controls (56)	Cluster-2.1 NPI (18)	Cluster-2.2 IPS (44)	Cluster-2.3 IPS-CMD (36)	Between group difference	Control vs NPI	Control vs IPS		Control vs IPS-CMD	NPI vs IPS	NPI vs IPS-CMD		IPS vs IPS-CMD
Cervical musculoskeletal impairments													
AROM flexion, mean ° (SD)	62.9 (9.7)	59.5 (10.7)	60.8 (11.1)	46.7 (9.5)	*F* = 13.9, *p* < 0.001*	*p* = 1.000	*p* = 0.456		*p* < 0.001*	*p* = 1.000	*p* = 0.004*		*p* < 0.001*
AROM extension, mean ° (SD)	72.9 (15.5)	65.8 (12.0)	71.3 (11.0)	56.2 (10.3)	*F* = 10.3, *p* < 0.001*	*p* = 0.593	*p* = 1.000		*p* < 0.001*	*p* = 1.000	*p* = 0.079		*p* = 0.001*
AROM right lateral flexion, mean ° (SD)^†^	40.0 (9.5)	41.2 (5.8)	42.8 (9.5)	30.9 (7.1)	*F* = 10.7, *p* < 0.001*	*p* = 1.000	*p* = 1.000		*p* < 0.001*	*p* = 1.000	*p* < 0.001*		*p* < 0.001*
AROM left lateral flexion, mean ° (SD)	46.1 (9.5)	43.2 (7.8)	46.1 (9.7)	31.6 (7.3)	*F* = 17.8 *p* < 0.001*	*p* = 1.000	*p* = 1.000		*p* < 0.001*	*p* = 1.000	*p* < 0.001*		*p* < 0.001*
AROM right rotation, mean ° (SD)	68.6 (8.6)	69.0 (8.0)	71.4 (7.3)	58.2 (9.7)	*F* = 13.0 *p* < 0.001*	*p* = 1.000	*p* = 1.000		*p* < 0.001*	*p* = 1.000	*p* < 0.001*		*p* < 0.001*
AROM left rotation, mean ° (SD)	69.6 (8.5)	68.9 (9.4)	62.1 (8.9)	59.6 (8.9)	*F* = 9.8 *p* < 0.001*	*p* = 1.000	*p* = 1.000		*p* < 0.001*	*p* = 1.000	*p* = 0.011*		*p* < 0.001*
FRT, total, mean° (SD)	102.2 (11.2)	85.3 (15.0)	92.4 (19.9)	72.9 (15.9)	*F* = 24.6 *p* < 0.001*	*p* = 0.002*	*p* = 0.001*		*p* < 0.001*	*p* = 1.000	*p* = 0.034*		*p* < 0.001*
APS, mean mmHg (SD)^§^	27.7 (2.8)	24.1 (4.0)	23.8 (3.7)	21.9 (2.2)	*χ*^2^ = 101.3, *p* < 0.001*	*p* = 0.006*	*p* < 0.001*		*p* < 0.001*	*p* = 1.000	*p* < 0.001*		*p* < 0.001*
Total MTrPs, mean number (SD)	3.9 (3.6)	7.4 (3.0)	8.1 (3.3)	7.8 (3.4)	*F* = 17.2, *p* < 0.001*	*p* = 0.001*	*p* < 0.001*		*p* < 0.001*	*p* = 1.000	*p* = 1.000		*p* = 1.000
Total PAIVMs, mean number (SD)^§^	0.9 (1.4)	2.9 (1.5)	2.9 (1.4)	2.7 (1.4)	*χ*^2^ = 79.0, *p* < 0.001*	*p* < 0.001*	*p* < 0.001*		*p* < 0.001*	*p* = 1.000	*p* = 1.000		*p* = 1.000
Quantitative sensory testing													
WUR temporalis, mean (SD)^†^	1.4 (2.1)	1.5 (2.0)	2.0 (1.9)	1.4 (2.0)	*F* = 1.2 P = 0.320	*p* = 1.000	*p* = 0.678		*p* = 1.000	*p* = 1.000	*p* = 1.000		*p* = 0.577
MPT temporalis, mean g (SD)^†^	22.1 (18.5)	11.3 (18.1)	11.2 (14.4)	12.8 (14.8)	*F* = 6.2 P < 0.001*	*p* = 0.008*	*p* = 0.009*		*p* = 0.022*	*p* = 1.000	*p* = 1.000		*p* = 1.000
sPPT temporalis, mean kPa (SD)^†^	248.3 (98.1)	316.9 (57.1)	172.5 (51.1)	188.4 (70.3)	*F* = 15.2 *p* < 0.001*	*p* = 0.064	*p* < 0.001*		*p* = 0.003*	*p* < 0.001*	*p* < 0.001*		*p* = 1.000
sPPT UCS total, mean kPa (SD)^†^	511.0 (232.3)	634.2 (161.9)	343.7 (103.2)	390.1 (143.6)	*F* = 12.6 *p* < 0.001*	*p* = 0.207	*p* < 0.001*		*p* = 0.008*	*p* < 0.001*	*p* < 0.001*		*p* = 1.000
sPPT LCS total, mean kPa (SD)^†^	605.2 (263.5)	705.8 (207.8)	400.8 (149.0)	442.2 (173.2)	*F* = 11.1 *p* < 0.001*	*p* = 1.000	*p* < 0.001*		*p* = 0.001*	*p* < 0.001*	*p* = 0.001*		*p* = 1.000
dPPT total mean g (SD)^†^	7766.1 (2779.6)	6877.8 (2897.8)	4430.2 (2516.4)	5095.3 (3056.8)	*F* = 13.3 *p* < 0.001*	*p* = 1.000	*p* < 0.001*		*p* < 0.001*	*p* = 0.039*	*p* = 0.189		*p* = 1.000
sPPT second MCP, mean kPa (SD)^†^	328.2 (135.9)	435.6 (122.1)	245.9 (79.9)	240.5 (78.3)	*F* = 14.2 *p* < 0.001*	*p* = 0.014*	*p* = 0.002*		*p* = 0.004*	*p* < 0.001*	*p* < 0.001*		*p* = 1.000
MPT thenar eminence, mean g (SD)^†^	32.3 (16.1)	30.7 (17.3)	22.0 (15.4)	20.8 (13.2)	*F* = 5.2 *p* = 0.002*	*p* = 1.000	*p* = 0.015*		*p* = 0.005*	*p* = 1.000	*p* = 0.550		*p* = 1.000
sPPT tibialis muscle, mean kPa (SD)^†^	438.3 (211.4)	638.9 (142.4)	358.5 (157.8)	357.9 (141.1)	*F* = 7.6 *p* < 0.001*	*p* = 0.005*	*p* = 0.353		*p* = 1.000	*p* < 0.001*	*p* < 0.001*		*p* = 1.000

*APS* activation pressure score, *AROM* active range of motion, *dPPT* dynamic pressure pain threshold, *FRT* flexion rotation test, *LCS* lower cervical spine, *MCP* metacarpophalangeal joint, *MPT* mechanical pain threshold, *MTrPs* myofascial trigger points, *PAIVMs* passive accessory intervertebral movements, *SD* standard deviation, *sPPT* static pressure pain threshold, *UCS* upper cervical spine, *WUR* wind-up ratio

^†^Data were log-transformed for statistical analysis (ANCOVA)

^‡^Data were square-root transformed for statistical analysis (ANCOVA)

^§^Data were treated as counting variable in a Poisson regression model

^*^Significant at *p* < 0.05

### Cohort 2: interictal migraine patients

#### Clinical characteristics

Cluster-2.3 (IPS-CMD) had the following:

- Compared to Cluster-2.1 (NPI): worse HDI-E (*p* = 0.010), NDI-physical (*p* = 0.009), and CSI (*p* = 0.018).
- Compared to Cluster-2.2 (IPS): longer disease duration (*p* = 0.006), higher headache frequency (*p* = 0.006), more use of drugs (*p* = 0.009), worse HDI-P (*p* = 0.003), HDI-E (*p* < 0.001), and HADS-D (*p* = 0.027)

No other differences were observed (Table 2b).

#### Psychophysical characteristics

Cluster-2.3 (IPS-CMD) had the following:

- Compared to controls: reduced AROM in flexion, extension, right and left lateral flexion, right and left rotation, FRT, APS, and higher number of MTrPs and PAIVMs (all, *p* < 0.001). Lower trigeminal MPT and sPPT, sPPT over upper and lower cervical spine, cervical dPPT, hand sPPT and MPT (all, *p* < 0.023).
- Compared to Cluster-2.1 (NPI): reduced AROM in flexion, right and left lateral flexion, right and left rotation, FRT, and APS (all, *p* < 0.034). Lower trigeminal sPPT, sPPT over upper and lower cervical spine, hand and leg sPPTs (all, *p* < 0.001).
- Compared to Cluster-2.2 (IPS): reduced AROM in flexion, extension, right and left lateral flexion, right and left rotation, FRT, APS (all, *p* < 0.001).

Cluster-2.2 (IPS) had the following:

- Compared to controls: reduced FRT, APS, and higher number of MTrPs and PAIVMs (all, *p* < 0.001). Lower trigeminal MPT and sPPT, sPPT over upper and lower cervical spine, cervical dPPT, hand sPPT and MPT (all, *p* < 0.015).
- Compared to Cluster-2.1 (NPI): lower trigeminal sPPT, sPPT over upper and lower cervical spine, cervical dPPT, hand and leg sPPTs (all, *p* < 0.039).

Cluster-2.1 (NPI) had compared to controls the following:

• Reduced FRT, APS, and higher number of MTrPs and PAIVMs (all, *p* < 0.006). Lower trigeminal MPT (higher sensitization) and higher hand and leg sPPTs (lower sensitization) (all, *p* < 0.014).

## Discussion

This paper results confirmed the existence of distinct migraine subgroups and associated phenotyping based on fourteen clinical PROMs and psychophysical bedside tools performed at different phases of the migraine cycle. These distinct migraine phenotypes showed significant differences in clinical presentations, somatosensory functions, and cervical musculoskeletal impairments, and further clinical importance is to be explored. As migraine patients with worse clinical and psychophysical characteristics also had worse psychological burden, a biopsychosocial approach should be considered in these migraine patients [14].

Moreover, these results added important information to what was found in a study just published by our research group [13]. First, even if most migraine patients (80%) showed increased pain sensitivity, 20% of them had reduced pain sensitivity (hypoalgesia) compared to healthy controls. Secondly, facilitation of the trigeminal temporal summation of pain seems to be a characteristic peculiar to ictal migraine patients, independent of the presence of increased pain sensitivity [15]. Third, subtle cervical musculoskeletal impairments seem to be present in all migraine subgroups compared to healthy subjects, strengthening the link existing between these physical characteristics and migraine pathophysiology. Fourth, even with different frequencies, all migraine subgroups had neck pain, suggesting that distinct mechanisms could underlie neck pain in the migraine population. In patients with no increased pain sensitivity but subtle cervical musculoskeletal impairments, neck pain is likely driven by peripheral mechanisms. On the other hand, in patients with increased pain sensitivity and cervical musculoskeletal impairments, both peripheral and central mechanisms could exist. Finally, in interictal migraine patients, impaired cervical musculoskeletal functionality, more than increased pain sensitivity, seems to be correlated with worse clinical characteristics and physiological burden.

### Migraine subgroups during the ictal/perictal phase

#### Clinical characteristics

Migraine patients with perictal/ictal increased pain sensitivity and cervical musculoskeletal dysfunctions (Cluster-1.2, IPS-CMD) had worse clinical characteristics compared to patients with no psychophysical impairment (Cluster-1.1, NPI) [35–37] confirming that this subgroup is worse affected by the disease. Cluster-1.2 (IPS-CMD) had higher headache intensity and disability, worse neck-related disability, worse quality of life, higher symptoms related to sensitization, and worse psychological burden. Due to the study design, it was not possible to identify which relationship exists between increased pain sensitivity, cervical musculoskeletal impairments, worse clinical characteristics, and psychological burden. On one hand, the enhanced pain sensitivity and the reduction in cervical musculoskeletal functionality occurring during the migraine attack [15, 16, 38] could lead to higher headache intensity [39, 40]. This will, in turn, cause higher disability, worse quality of life, and higher psychological burden [37, 41, 42]. On the other hand, individual psychological and social factors can influence patients’ elaboration of the pain experience leading to a worse perception of the disease, increased pain sensitivity, higher headache intensity, and worse disability [14]. This attitude regarding the pain experience will, in turn, cause an avoidance behavior during the headache attack, impairing cervical musculoskeletal functionality [43]. Future longitudinal studies should assess the relationship existing between increased pain sensitivity, cervical musculoskeletal impairments, worse clinical characteristics, and psychological burden in migraine patients.

### Psychophysical characteristics

#### Cervical musculoskeletal impairments

This study results showed, using a comprehensive cervical musculoskeletal evaluation, that Cluster-1.2 (IPS-CMD) had worse cervical musculoskeletal impairments compared to Cluster-1.1 (NPI) and healthy controls. As Cluster-1.2 also had increased widespread pain sensitivity, a relationship between musculoskeletal impairments and pain sensitivity seems to occur in migraine patients in proximity and during a headache attack [44, 45]. Even if patients in Cluster-1.2 (NPI) had no reduction of cervical active and passive mobility, they showed worse functionality of deep cervical flexor muscles and a higher number of myofascial and articular areas that can reproduce referred pain compared to healthy subjects. The reduction in deep cervical flexor muscle functionality was observed in both migraine subgroups compared to controls, without difference between Cluster-1.1 (NPI) and Cluster-1.2 (IPS-CMD). These results support that the craniocervical flexion test could differentiate migraine patients compared to healthy subjects but did not seem to have a direct relationship with worse clinical characteristics in migraine patients [46]. On the other hand, Cluster-1.1 (NPI) had a higher number of myofascial and articular areas that can reproduce referred pain compared to controls, but lower compared to Cluster-1.2 (IPS-CMD). Thus, due to their higher prevalence, an increased number of Myofascial TrPs and positive cervical vertebral segments could be a characteristic common to most migraine patients [31, 47]. However, they seem to be more enhanced in Cluster-1.2 (IPS-CMD), suggesting a relationship with worse clinical characteristics in migraine patients [16].

#### Somatosensory function

Using a comprehensive quantitative sensory testing protocol, the data confirmed increased trigeminal, cervical, and widespread pain sensitivity during the migraine attack occurred in 81% of the migraine population (IPS-CMD) [38]. The remaining 19% of patients (NPI) showed higher QST value (reduced pain sensitivity) compared to controls, suggesting that the increased pain sensitivity that characterized the migraine attack [38, 48] did not occur in all migraineurs, with a subgroup of patients showing signs of reduced pain sensitivity [49].

However, temporal summation of pain in the trigeminal area was facilitated in both migraine clusters and was peculiar to the ictal phase [15]. As WUR was increased also in patients with reduced pain sensitivity, different mechanisms could underlie the facilitation in temporal summation of pain and the increase in pain sensitivity [50, 51].

Increased trigeminal, cervical, and widespread pain sensitivity could be a sign of pain amplification in the trigeminocervical complex and subcortical/cortical brain areas [38, 52]. On the other hand, the facilitation of trigeminal temporal summation of pinprick pain could reflect enhanced gain in the trigeminocervical complex [52]. Both presynaptic and postsynaptic sensitization mechanisms and impairment of descending pain modulation [53] mechanisms could facilitate the trigeminal temporal summation of pain [54]. As WUR was also facilitated in migraine patients with reduced pain sensitivity in the trigeminocervical area, who did not show signs of enhanced pain amplification at the trigeminocervical level, it is more likely that impairment of descending pain modulation mechanisms could account for the facilitation of trigeminal temporal summation of pain observed [53]. Future studies should assess the impairment of descending pain modulation mechanisms in migraine patients focusing on different phenotypes and migraine phases.

### Migraine subgroups during the interictal phase

#### Clinical characteristics

The migraine subgroup with interictal increased pain sensitivity and cervical musculoskeletal dysfunctions (Cluster-2.3, IPS-CMD) was worse affected by the disease, with worse headache and neck-related disability and higher symptoms of sensitization compared to patients with no psychophysical impairment (Cluster-2.1, NPI), and worse headache characteristics, disability, and psychological burden compared to patients with increased pain sensitivity (Cluster-2.2, IPS).

In a previous paper, we hypothesize that, as they share similar prevalence, general, and clinical characteristics, Cluster-1.1 (NPI, ictal) and Cluster-2.1 (NPI interictal) could represent the same subgroup assessed in different migraine phases [55]. On the other hand, the remaining 80% of the sample will be represented by one group when patients were assessed ictally/preictally (Cluster-1.2 IPS-CMD), and by two different subgroups when patients were assessed interictally (Cluster-2.3 IPS-CMD and Cluster-2.2 IPS interictally). As Cluster-2.3 (IPS-CMD) had longer disease duration and higher headache frequency compared to Cluster-2.2 (IPS), these clusters could be considered a clinical continuum representing the two extremities in terms of disease progression of a single group assessed ictally/preictally (Cluster-1.2 IPS-CMD). This paper’s results seem to confirm this hypothesis using multiple questionnaires to assess clinical characteristics [55].

Interestingly, Cluster-2.3 (IPS-CMD) but not Cluster-2.2 (IPS), had worse neck-rerated disability compared to Cluster-2.1 (NPI). Moreover, even if it did not reach a statistical significance, Cluster-2.3 (IPS-CMD) had worse neck-related disability also compared to Cluster-2.2 (IPS). As Cluster-2.3 (IPS-CMD) and Cluster-2.2 (IPS) had signs of increased pain sensitivity, but only the former also had cervical musculoskeletal dysfunctions, not only “central sensitization” mechanisms [23, 56], but also peripheral mechanisms [57, 58], could influence the presence and the burden of neck pain in migraine patients.

### Psychophysical characteristics

#### Cervical musculoskeletal impairments

When migraine patients were assessed in the interictal phase, one migraine subgroup (Cluster-2.3, IPS-CMD) had worse cervical musculoskeletal impairments with reduced active and passive cervical mobility and worse functionality of deep cervical flexor muscles compared to matched healthy controls, to Cluster-2.1(NPI), and to Cluster-2.2(IPS). As Cluster-2.3(IPS-CMD) was the subgroup worse affected by the disease, a relationship seems to occur between cervical musculoskeletal functionality and disease burden [16, 59], outlining the importance to assess and address cervical musculoskeletal dysfunctions in migraine patients.

As reduced cervical passive mobility and deep cervical flexor muscles’ functionality, and higher areas that reproduce referred pain, were also observed in Cluster-2.1 (NPI) and Cluster-2.2 (IPS) compared to controls, subtle cervical musculoskeletal impairments could occur independently by the presence of increased pain sensitivity [56] and the clinical manifestation of the disease [46]. On the other hand, reduced cervical active range of motion seems to be peculiar to a subgroup with worse clinical characteristics [16, 59], increased pain sensitivity, and further impairment of cervical musculoskeletal functionality. Thus, to enhance the clinical validity of the cervical musculoskeletal assessment, clinicians should consider the degree of cervical musculoskeletal impairments. Cervical active mobility should be considered a key driver to evaluate the extent of cervical musculoskeletal impairments.

#### Somatosensory function

Cluster-2.2 (IPS) and 2.3 (IPS-CMD) had increased trigeminal, cervical, and widespread pain sensitivity assessed with multiple QST compared to Cluster-2.1 (NPI) and controls. On the other hand, like Cluster-1.1 (NPI preictal/ictal), Cluster-2.1 (NPI interictal) had reduced pain sensitivity compared to controls supporting that these two subgroups could represent the same cluster assessed in different migraine phases. Therefore, about 20% of migraine patients showed reduced pain sensitivity independently by the headache phase [49].

As Cluster-2.2 (IPS) and 2.3 (IPS-CMD) had a similar somatosensory profile to Cluster-1. 2(IPS), they could be considered two subgroups of the same cluster assessed in preictal/ictal phase (Cluster-1.2 IPS).

If activity-dependent sensitization mechanisms could account for the preictal/ictal increased pain sensitivity, late-onset transcription-dependent sensitization mechanisms seem to account for the interictal increased pain sensitivity [52]. The fact that, among the 80% of migraine patients with long-lasting increased pain sensitivity, only a subgroup of patients with a longer disease duration also had cervical musculoskeletal dysfunctions suggested that late-onset transcription-dependent sensitization mechanisms protracted over time could affect cervical motor behavior [44, 45]. However, future longitudinal studies should assess the bidirectional relationship between increased pain sensitivity and cervical musculoskeletal dysfunction in migraine patients.

### Limitation

The population was recruited from specialized headache centers, and over two-third of the patients were excluded for age, concomitant pathologies, and concomitant diagnosis of other headache types. Thus, the external validity of these results should be interpreted with caution.

Development of pain sensitization is a time-dependent phenomenon that gradually develops approaching the migraine attack [17, 60]. Therefore, the inclusion of perictal and ictal migraine patients and the lack of controlling for the distance from the last/next headache attack performed in cohort 1 could have biased the results. However, in the previous paper in which different subgroups were defined, it was shown that the two clusters observed in cohort 1 did not differ in the headache phase or hours from the last/next headache attack [13].

## Conclusion

Distinct migraine subgroups identified in different migraine phases by a set of clinical PROMs and quantitative bedside assessment tools could be considered clinically different migraine phenotypes. These distinct phenotypes showed different clinical and psychophysical characteristics assessed by multiple clinical-related questionnaires, comprehensive quantitative sensory testing, and cervical musculoskeletal assessment. Some specific parameters such as increased disability, lower cervical active range of motion, and reduced pressure pain thresholds were specifically sensitive to separate the distinct phenotypes. The clinical implications of the different phenotypes may provide different responses to migraine treatments and need further exploration.
